# Study on correlations of BDNF, PI3K, AKT and CREB levels with depressive emotion and impulsive behaviors in drug-naïve patients with first-episode schizophrenia

**DOI:** 10.1186/s12888-023-04718-8

**Published:** 2023-04-03

**Authors:** Shan Li, Cailian Lu, Lin Kang, Qianqian Li, Hongxu Chen, Han Zhang, Ziling Tang, Yanwen Lin, Meiyan Bai, Peng Xiong

**Affiliations:** 1 Department of Psychiatry, First Affiliated Hospital of Kunming Medical University, Kunming, China; 2 Yunnan Clinical Research Center for Mental Disorders, First Affiliated Hospital of Kunming Medical University, Kunming, China

**Keywords:** Schizophrenia, Depressive emotion, Impulsive behavior, Serum protein factor

## Abstract

**Background:**

The pathogenesis of schizophrenia is still unknown. Nearly a half of schizophrenic patients have depressive symptoms and even some impulsive behaviors. The definite diagnosis of schizophrenia is an immense challenge. Molecular biology plays an essential role in the research on the pathogenesis of schizophrenia.

**Objective:**

This study aims to analyze the correlations of serum protein factor levels with depressive emotion and impulsive behaviors in drug-naïve patients with first-episode schizophrenia.

**Methods:**

Seventy drug-naïve patients with first-episode schizophrenia and sixty-nine healthy volunteers from the health check center in the same period participated in this study. In both the patient group and control group, brain-derived neurotrophic factor (BDNF), phosphatidylin-ositol-3-kinase (PI3K), protein kinase B (AKT), and cAMP-response element binding protein (CREB) levels in the peripheral blood were tested by enzyme-linked immunosorbent assay (ELISA). The depressive emotion and impulsive behaviors were evaluated with Chinese versions of the Calgary Depression Scale for Schizophrenia (CDSS) and Short UPPS-P Impulsive Behavior Scale (S-UPPS-P), respectively.

**Results:**

The serum levels of BDNF, PI3K, and CREB in the patient group were lower than those in the control group, while AKT level, total CDSS score and total S-UPPS-P score were all higher. In the patient group, total CDSS score, and total S-UPPS-P score were both correlated negatively with BDNF, PI3K, and CREB levels but positively with AKT level, and the lack-of-premeditation (PR) sub-scale score was not significantly correlated with BDNF, PI3K, AKT, and CREB levels.

**Conclusion:**

Our study results showed that the peripheral blood levels of BDNF, PI3K, AKT, and CREB in drug-naïve patients with first-episode schizophrenia were significantly different from those in the control group. The levels of these serum protein factors are promising biomarkers to predict schizophrenic depression and impulsive behaviors.

## Introduction

The etiology of schizophrenia has not been clarified yet. Schizophrenia is characterized by positive, negative, cognitive, behavioral, and emotional symptoms, has a significant impact on the social and occupational functions of patients, and its lifelong morbidity is 0.30–0.66% [1]. Schizophrenia is a heavy burden for patients families and the whole of society. In clinical practice, psychiatrists pay more attention and importance to positive, negative, and cognitive symptoms, but the incidence rate of depression is high in various stages of schizophrenia. A domestic study demonstrated that 49.37% of patients with first-episode schizophrenia presented depressive symptoms [2]. There is a higher incidence rate of depression in elderly patients with schizophrenia spectrum disorders (SSD). A cross-sectional study showed that depression had a morbidity of up to 78.1% in this population [3], Another longitudinal study suggested that 2/5 of these patients presented permanent depression, and 1/4 might be fluctuating between depression and non-depression [4]. Suicide is a serious adverse outcome of depression, and the lifelong suicide rate of schizophrenic patients is estimated as 5 folds of that of ordinary populations [5]. A meta-analysis revealed that there was no significant correlation between the tendency and incidence rate of suicide in patients with emotional disorders, but the incidence rate of suicide was increased by ≥ 6 folds in SSD patients with a suicide tendency [6]. Considering the extremely high suicide risk in schizophrenic patients with depressive symptoms, it is very important to determine the risk factors that can be used for effective screening and prevention in this population. Similarly, an impulse is not a unique symptom of schizophrenic patients, but successive studies are showing increased impulsive behaviors in these patients [7]. Due to impulse control impairment, schizophrenic patients may have an accordingly increased risk of violent behaviors [8], suicide [9], and substance abuse [10] may follow. Therefore, it is an important treatment goal for schizophrenia to reduce impulsive behaviors.

**Fig. 1:**
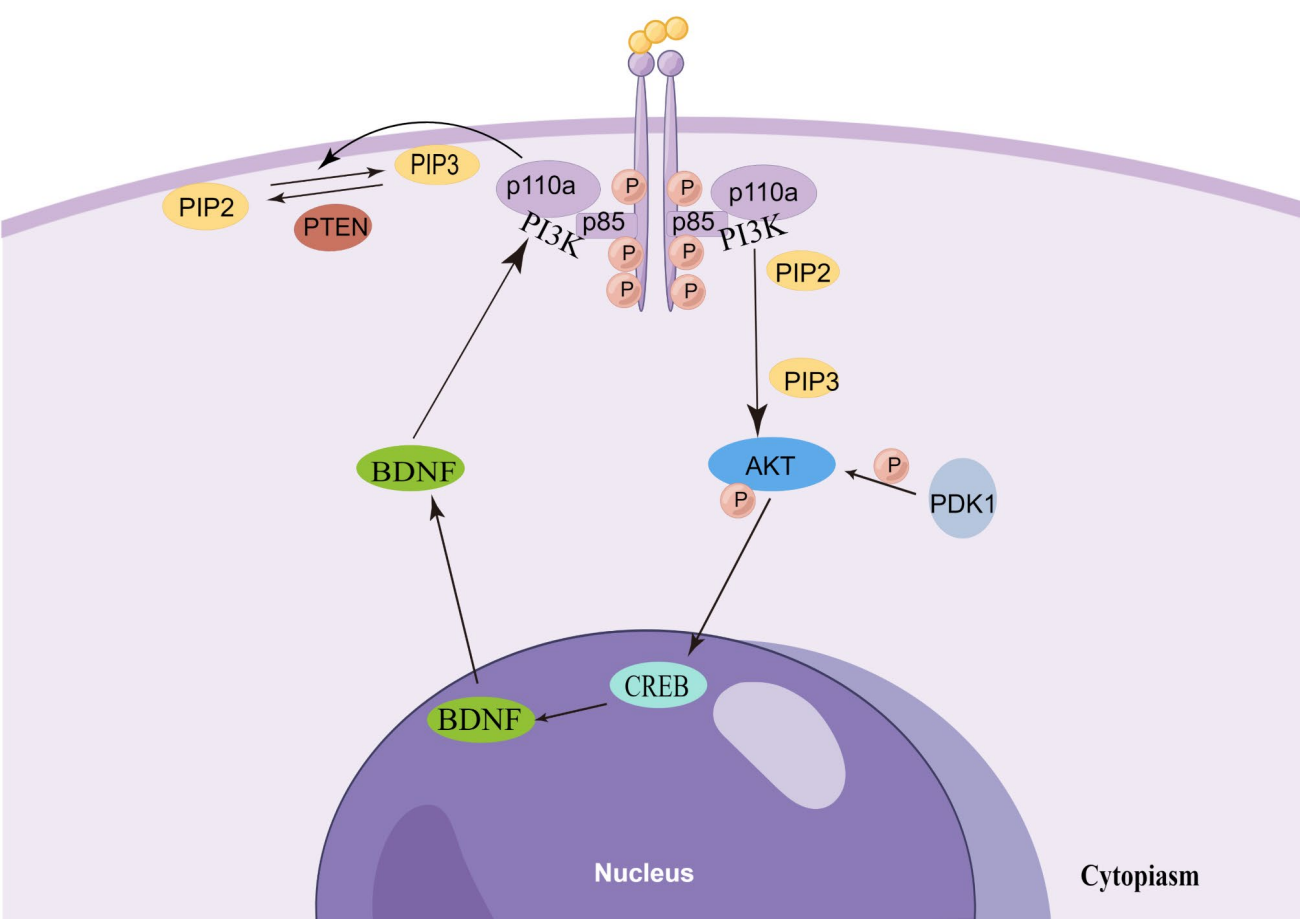
Based on the measured serum proteins, we drew their pathway hypothesis (by Figdraw). (BDNF, brain-derived neurotrophic factor; PI3K, phosphatidylinositol-3 kinase; AKT, protein kinase B; CREB, cAMP response element binding protein; PIP2, phosphatidylinositol 4,5-biphosphate; PIP3, phosphatidylinositol 3,4,5-trisphosphate; PTEN, phosphatase and tensin homolog; PDK1, Phosphoinositide dependent kinase 1.)

A part of previous studies revealed the clinical risk factors and possible pathophysiological mechanisms of depression and impulse in schizophrenia. The depressive symptoms of schizophrenic patients are associated with an early onset age, serious negative symptoms, serious common psychotic symptoms, akathisia, dyskinesia [11], cognitive impairment [2], high serum LDL and TC levels [12], and even insomnia [13]. Schizophrenic impulse is related to frontotemporal circuit dysfunction [14] and cortical thickness [15]. At present, there are few studies about the pathophysiological mechanism of depressive symptoms and impulsive behaviors in drug-naïve patients with first-episode schizophrenia. Thus, considering some differential pathophysiological changes in different disease phases and possible drug & treatment effects, it is critical to supplement the relevant studies. Our previous study has preliminarily proven the diagnostic values of several peripheral serum protein factors for schizophrenia [16, 17]. The pathophysiological mechanism of schizophrenia may be associated with serum protein factors concerning neurotrophy, synaptic transmission, and cellular signaling. BDNF plays a dual role in neurotrophy and synaptic growth [18], and it is a member of the neurotrophic factor family and regulates the proliferation, differentiation, survival, and death of neurons and gliacytes [19]. BDNF is a secretory polypeptide distributed in the central nervous system and other organs, and can penetrate the blood-brain barrier [20]. BDNF exhibits a trophic structure and physiological functions in the central nervous system via tropomyosin-related kinase B (TrkB) receptor, promotes the synaptic growth, plasticity, and connectivity of nerves and the survival of neurons, and prevents cell apoptosis [21]. Phosphorylated TrkB can activate three different signaling pathways (Mitogen-activated protein kinase (MAPK), PI3K, and phospholipase C-γ (PLC-γ)) and thus promote neurogenic, synaptogenic, and other activities [22]. PI3K is an important kinase of inositol and phosphoinositol and a dimer consisting of a 110 kDa catalytic subunit and an 85 kDa regulatory subunit, and can regulate cell growth and migration. PI3K phosphatidylinositol 4,5-bisphosphate (PIP2) produces phosphatidylinositol [3–5]-trisphosphate (PIP3) as the second messenger. Subsequently, AKT is recruited onto the internal surface of the plasma membrane by PIP3 and then phosphorylated and activated via phosphoinositide-dependent protein kinase 1 (PDK1). PI3K/AKT pathway participates in synaptic transmission and nerve plasticity by forming an intracellular central network through the transduction of cell survival signals [23], and it plays an important role in cell growth, proliferation, survival, and differentiation. According to some study reports, BDNF works in the development of schizophrenia, Parkinson’s disease, and Alzheimer’s disease by binding with TrkB and then activating PI3K/AKT signaling pathway [24]; CREB plays a key role in the occurrence and development of schizophrenia, perhaps associated with the regulation of CREB on BDNF expression [25, 26] ; The phosphorylation of upstream PI3K and its effector AKT can stimulate CREB activation in neurons [27]. Therefore, we proposed a hypothesis that the pathophysiological mechanism of depression and impulsive behaviors in drug-naïve patients with first-episode schizophrenia might be associated with BDNF, PI3K, AKT, and CREB in the corresponding cellular signaling pathways. (See Fig. 1) Some animal studies showed that mice with BNDF defect might have depressive manifestations [28]. A recent study demonstrated that BDNF level in the peripheral blood was lower in schizophrenic patients with depressive symptoms than in those without such symptoms [29]. In another longitudinal study, the depressive symptoms of schizophrenic patients were relieved by elevating the serum BDNF level after drug treatment [30]. BDNF has a certain correlation with the impulsive behaviors of patients with mental disorders, and it was proposed as a possible biomarker in some studies [31]. In the animal experiment, PI3K/AKT signaling pathway participated in regulating neuroinflammation and neurotrophy, thus affecting the depressive manifestations of mice [32]. A study of human mental disorders demonstrated that the etiology of depressive emotion was also related to the regulation of PI3K/AKT signals [23]. In addition, AKT absence could induce anxiety, depressive emotion, and behavioral changes [33]. CREB was involved in transcriptional regulation [34] and associated with impulse regulation [35, 36]; Some studies showed that CREB expression was decreased in the cortex of patients with severe depression [37]. Based on the previous study results abovementioned, four proteins (BDNF, PI3K, AKT, and CREB) in this study did not work independently in regulating the normal functions of cells, and they linked with each other frequently to form the pathways. Therefore, when validating the effects of these four proteins on depressive emotions and impulsive behaviors in schizophrenia, we further explored the formation potential of protein-protein pathways. In the present study, we first tested the serum levels of BDNF, PI3K, AKT, and CREB related to neurotrophy, synaptic transmission, and cellular signaling in drug-naïve patients with first-episode schizophrenia, and then analyzed their correlations with schizophrenic depressive emotion and impulsive behaviors using more objective data. This study is expected to provide convenience and reference for the prevention and treatment evaluation of different clinical characteristics of schizophrenia.

## Methods

### Participants

The drug-naïve patients with first-episode schizophrenia were included and were admitted to the Psychiatry Department of the First Affiliated Hospital of Kunming Medical University from October 2020 to May 2022. The inclusion criteria were as follows: ①Patients who met the diagnostic criteria of schizophrenia in the International Statistical Classification of Diseases and Related Health Problems 10th Revision (ICD-10); ② Male or female patients at an age of 18–60 years; ③ Patients with first-episode schizophrenia who did not use antipsychotic drugs; ④ Patients who could cooperatively complete CDSS and S-UPPS-P examinations; ⑤ Patients who could cooperatively complete PANSS examination and had a total PANSS score ≥ 60; and ⑥ Patients who or whose families signed the informed consent forms. The exclusion criteria are the following: ① Patients with non-schizophrenic mental disorders such as mental retardation, depressive disorders, anxiety disorders, and substance dependence; ② Patients with a history of epilepsy, encephalitis, and other cerebral organic diseases or nervous system diseases; ③ Patients with diseases of the immune system, endocrine system or other systems; ④ Patients with the abuse of drugs or psychoactive substances; ⑤ Patients with a history of craniocerebral trauma; ⑥ Patients with a history of drug allergy, hormone treatment or immunosuppressant treatment; ⑦ Pregnant or breast-feeding women; and ⑧ Patients who had an apparent impulse or were not cooperative. The healthy volunteers from the health check center served as the control group. The inclusion criteria were as follows: ① Patients who denied a history of mental disorders, a familial history of mental disorders, and a history of chronic diseases of the immune system; ② Male or female patients at an age of 18–60 years; and ③ Patients who had good compliance and could cooperatively complete the relevant examinations. The exclusion criteria were the same as those for the patient group. Due to poor cooperation of partially enrolled patients in the scale examinations and subsequently improper storage & processing of blood samples, 6 and 2 subjects were rejected separately in the patient group and control group, and eventually, there were 70 cases (43 males and 27 females) in the patient group and 69 cases (35 males and 34 females) in the control group. Regards to gender, smoking, BMI, and education, there was no statistically significant difference between the two groups (χ²=1.617, P = 0.234; χ²=2.966, P = 0.109; Z=-0.613, P = 0.540; Z=-0.729, P = 0.466). Our study was approved by the Ethics Committee of the First Affiliated Hospital of Kunming Medical University, and the patients and volunteers included in this study or their families all signed the informed consent forms.

### Sample collection and measurement of biochemical indicators

In the next morning after enrollment, 5mL venous blood was collected into the pro-anticoagulation tube via the ulnar vein in the patient group, preserved at room temperature for 30 min, and then centrifuged at 3,000×g for 15 min, and the serum of each blood sample was collected and put into 4 tubes (200ul/tube). The test serum samples were preserved at the − 80 ℃ refrigerator. After the samples were all collected at different time points, the serum levels of BDNF, PI3K, AKT, and CREB were synchronously tested by enzyme-linked immunosorbent assay (ELISA) at the same time. The collection and storage of serum samples in the control group were finished using the same methods as the patient group, and the testing of BDNF, PI3K, AKT, and CREB levels was synchronously performed in the same period. Each serum sample was tested twice, and two test results were averaged to give the final result. The same batch of reagent kits and supporting reagents produced by Shanghai Enzyme-linked Biotechnology Co., Ltd. were used.

### Clinical evaluation

Evaluation of depressive emotion: The depressive symptoms of subjects were evaluated by two professionally trained psychiatrists with the conformity certificate using the Chinese version of the Calgary Depression Scale for Schizophrenia (CDSS) [38], and a total score of ≥ 6 indicated significant depressive symptoms. Evaluation of Impulsive Behaviors: The impulsive behaviors of subjects were evaluated by the above-mentioned two physicians using the Chinese version of the Short UPPS-P Impulsive Behavior Scale (S-UPPS-P) [39]. The scale contains 5 impulse subscales: Negative urgency (NU), positive urgency (PU), sensation seeking (SS), lack of premeditation (PR), and lack of perseveration (PE).

### Data analysis

Statistical Processing: SPSS 25.0 statistic software was used for statistical analysis. Shapiro-Wilk test was used for normality test of all measurement data. The normal distribution data were expressed as mean standard deviation (χ ± s), and the non-normal distribution data were expressed as median interquartile(M ± Q). Independent sample t-test was used to test the variables that conformed to normal distribution, and Mann-Whitney U-test was used to compare the variables that did not conform to normal distribution among groups. The intergroup comparisons of categorical data were performed using the χ² test. Correlations between serum protein factor concentrations were analyzed using either Pearson (normal distribution variable) or Spearman (non-normal distribution variable) correlations. Spearman (non-normal distribution variable) correlation analysis was used to explore the relationship between total CDSS score, total S-UPPS-P score, and S-UPPS-P subscale scores in patients. In view of the significant correlation found between protein concentrations in the patient group, to avoid multicollinearity, we first performed principal component analysis on the four serum protein factors. In the patient group, with total CDSS score, total S-UPPS-P score, and S-UPPS-P subscale scores as dependent variables, as well as Serum protein factor principal component value F, gender, marital status, age, BMI, smoking, and education as independent variables, multivariate linear regression analysis was performed to investigate the influential factors of depressive emotions, impulsive behaviors, and various subscale scores. P < 0.05 indicated a statistically significant difference.

## Results

### Demographic data

Table 1 shows the demographic data of the patient group (n = 70) and the healthy control group (n = 69). There were no statistically significant differences in the gender, smoking, education, BMI, and total PANSS score (P > 0.05) but statistically significant differences in the age and marital status between the two groups (P < 0.05). These data would be used for subsequent analysis.

**Table 1:** Descriptive statistics of schizophrenia patients and healthy controls

	Patients	Controls	t/Z/χ²	P
	(n = 70)	(n = 69)		
Gender(male/female, n)^a^	43/27	35/34	1.617	0.234
Marital status(single/married, n)^a^	57/13	22/47	34.768	0.000
smoking(yes/no, n) ^a^	29/41	19/50	2.966	0.109
Age(year)^b^	25.00 ± 14.30	32.00 ± 12.50	-3.738	0.000
Education (years)^b^	12.00 ± 7.00	12.00 ± 5.50	-0.729	0.466
BMI(kg/m²)^b^	21.48 ± 3.52	20.96 ± 3.22	-0.613	0.540
Total PANSS score^b^	87.46 ± 5.77	54.00 ± 9.00	-10.180	0.000
Total CDSS score^b^	6.00 ± 8.00	1.00 ± 2.00	-7.234	0.000
Total S-UPPS-P score^c^	48.19 ± 8.41	37.07 ± 6.47	-8.724	0.000
S-UPPS-P				
NU subscore ^b^	11.00 ± 3.30	7.00 ± 4.00	-5.442	0.000
PU subscore ^b^	10.00 ± 4.30	9.00 ± 4.00	-2.820	0.005
SS subscore ^b^	10.00 ± 4.00	8.00 ± 4.00	-4.161	0.000
PR subscore ^b^	8.27 ± 2.76	6.00 ± 2.00	-5.482	0.000
PE subscore ^b^	8.97 ± 2.71	6.00 ± 2.50	-6.202	0.000
Levels of protein factor(x¯±s)				
BDNF(ng/mL) ^b^	3.64 ± 1.60	5.27 ± 1.51	-7.149	0.000
PI3K(ng/mL)^c^	3.95 ± 1.38	5.49 ± 2.07	5.146	0.000
AKT(umol/L)	10.79 ± 2.28 ^c^	8.14 ± 2.44 ^b^	-6.102	0.000
CREB(ng/mL) ^b^	1.95 ± 1.13	2.76 ± 1.38	-5.228	0.000

BMI, body mass index; PANSS, Positive and Negative Syndrome Scale; CDSS, Calgary Depression Scale for Schizophrenia; S-UPPS-P, The Short UPPS-P Impulsive Behavior Scale; NU, Negative urgency; PU, positive urgency; SS, sensation seeking; PR, lack of premeditation; PE, and lack of perseveration; BDNF, brain-derived neurotrophic factor; PI3K, phosphoinositide-3-kinase; AKT, protein kinase B; CREB, cyclic AMP response-element binding protein. The data were expressed as the number of cases or the average standard deviation or the median quartile interval. a, chi-square test; b, nonparametric test; c, t test.

### Serum BDNF, PI3K, AKT and CREB levels

Table 1 shows the serum levels of four protein factors in the patient group and control group. The results revealed that BDNF, PI3K and CREB levels in the patient group were significantly lower than those in the control group (Z=-7.149, P < 0.001; t = 5.146, P < 0.001; Z=-5.228, P < 0.001), while the AKT level was higher (Z=-6.102, P < 0.001).

Besides, the correlations between biomarkers were analyzed by Pearson or Spearman correlation analysis. Our results showed that in the patient group, BDNF, PI3K and CREB levels had mutually positive correlations, but the AKT level was in a negative correlation with the former three (P < 0.05), while no significant correlations were observed in the control group (See Table 2).

**Table 2:** Correlation analysis of the association between BDNF, PI3K, AKT and CREB concentrations

	patients			controls	
	r/rS	P	rS		P
BDNF&PI3K	0.943 ^rS^	0.000	0.220		0.070
BDNF&AKT	-0.958 ^rS^	0.000	-0.090		0.461
BDNF&CREB	0.944 ^rS^	0.000	0.010		0.935
PI3K&AKT	-0.970 ^r^	0.000	-0.021		0.865
PI3K&CREB	0.865 ^rS^	0.000	0.006		0.963
AKT&CREB	-0.881 ^rS^	0.000	0.152		0.212

BDNF, brain-derived neurotrophic factor; PI3K, phosphoinositide-3-kinase; AKT, protein kinase B; CREB, cyclic AMP response-element binding protein. r, Pearson correlation analysis was used; rS, Spearman correlation analysis was used.

### Principal component analysis of serum levels of protein factors

In the patient group, the applicability test of factor analysis shows that Kaiser-Meyer-Olkin test results in KOM value of 0.812, Bartlett spherical test results in 512.648, and Sig value of 0.000, indicating that the data of protein factor concentration is suitable for factor analysis. Determination of initial factor load by principal component analysis. As can be seen from Table 3, the output result of the principal component method is to extract one principal component, F1. The initial eigenvalue of the principal component totals 3.792, and the cumulative contribution rate is 94.798%. This shows that the extracted principal component can explain 94.798% of the four protein indexes, and fully retain the information of the original variables, and the extracted principal component is representative. Combined with the component matrix of the principal component (Table 4), the coefficients of BDNF, PI3K, AKT and CREB are calculated. Coefficient of BDNF = $\frac{0.988}{\sqrt{3.792}}$, coefficient of PI3K =$\frac{0.972}{\sqrt{3.792}}$, coefficient of AKT =$\frac{-0.981}{\sqrt{3.792}}$, coefficient of CREB =$\frac{0.953}{\sqrt{3.792}}$. The principal component equations of four serum protein factors are further obtained as follows: F = 0.507×BDNF+0.499×PI3K-0.504×AKT+0.489×CREB.

**Table 3:** Principal component results of BDNF, PI3K, AKT and CREB concentrations in patients

total variance explanation						
component	Initial eigenvalue			Extracting sum of squares of loads		
	total	variance %	accumulate %	total	variance %	accumulate %
1	3.792	94.798	94.798	3.792	94.798	94.798
2	0.151	3.768	98.566			
3	0.031	0.780	99.346			
4	0.026	0.654	100.000			

BDNF, brain-derived neurotrophic factor; PI3K, phosphoinositide-3-kinase; AKT, protein kinase B; CREB, cyclic AMP response-element binding protein.

**Table 4:** Component matrix of protein concentration principal component in patients

	principal constituent 1
BDNF	0.988
PI3K	0.972
AKT	-0.981
CREB	0.953

### Symptoms and serum levels of protein factors

The results of Spearman correlation analysis showed that there was no significant correlation between total CDSS score and total S-UPPS-P score and its five subscale scores in the patient group (all P > 0.05) (See Table 5).

**Table 5:** Correlation between total CDSS score and total S-UPPS-P score and its five subscale scores in patients

	CDSS & S-UPPS-P	CDSS & N U	CDSS & PU	CDSS & SS	CDSS & PR	CDSS & PE	
rS	0.074	0.101	0.113	0.133	0.031	0.014	
P	0.543	0.406	0.354	0.274	0.801	0.908	

CDSS, Calgary Depression Scale for Schizophrenia; S-UPPS-P, The Short UPPS-P Impulsive Behavior Scale; NU, Negative urgency; PU, positive urgency; SS, sensation seeking; PR, lack of premeditation; PE, and lack of perseveration; rS, Spearman correlation analysis was used.

In the patient group, multivariate linear regression analysis was performed with total CDSS score, total S-UPPS-P score, and S-UPPS-P subscale scores as dependent variables, as well as the principal component value F, gender, marital status, age, BMI, smoking, and education as independent variables. The results showed that the principal component value F was the influential factors of total CDSS score, total S-UPPS-P score, and S-UPPS-P NU, PU, SS & PE subscale scores (P < 0.05), but it had no significant correlations with PR subscale score (P>0.05). Furthermore, marital status and age might be influential factors in the PR subscale score (P < 0.05), and age might be an influential factor in the PE subscale score (P < 0.05) together with the principal component value F (See Tables 6, 7, 8, 9, 10, 11 and 12).

**Table 6:** Multivariate regression analysis of total CDSS score and influencing factors in patients

	B	SE	β	t	P
F value	-0.891	0.280	-0.395	-3.180	0.002
age	0.025	0.050	0.064	0.496	0.622
BMI	-0.165	0.208	-0.096	-0.795	0.430
Education	0.149	0.158	0.111	0.943	0.349
Gender	-0.490	1.183	-0.055	-0.414	0.680
Marital status	-0.344	1.382	-0.031	-0.249	0.804
smoking	0.814	1.181	0.092	0.689	0.494

CDSS, Calgary Depression Scale for Schizophrenia; F value, the principal component value of BDNF, PI3K, AKT and CREB; BMI, body mass index.

**Table 7:** Multivariate regression analysis of total S-UPPS-P score and influencing factors in patients

	B	SE	β	t	P
F value	-2.595	0.470	-0.601	-5.525	0.000
age	-0.077	0.083	-0.105	-0.927	0.358
BMI	0.236	0.348	0.072	0.677	0.501
Education	-0.010	0.265	-0.004	-0.036	0.971
Gender	1.682	1.983	0.098	0.848	0.399
Marital status	0.320	2.316	0.015	0.138	0.891
smoking	2.267	1.980	0.134	1.145	0.257

S-UPPS-P, The Short UPPS-P Impulsive Behavior Scale; F value, the principal component value of BDNF, PI3K, AKT and CREB; BMI, body mass index.

**Table 8:** Multivariate regression analysis of NU subscale score and influencing factors in patients

	B	SE	β	t	P
F value	-0.770	0.168	-0.519	-4.581	0.000
age	-0.039	0.030	-0.156	-1.321	0.191
BMI	0.203	0.125	0.180	1.632	0.108
Education	0.001	0.095	0.001	0.006	0.995
Gender	0.244	0.709	0.041	0.344	0.732
Marital status	-0.474	0.828	-0.064	-0.572	0.570
smoking	0.992	0.708	0.170	1.401	0.166

NU, Negative urgency; F value, the principal component value of BDNF, PI3K, AKT and CREB; BMI, body mass index.

**Table 9:** Multivariate regression analysis of PU subscale score and influencing factors in patients

	B	SE	β	t	P
F value	-0.569	0.199	-0.349	-2.864	0.006
age	0.037	0.035	0.134	1.060	0.293
BMI	0.229	0.147	0.184	1.550	0.126
Education	0.015	0.112	0.015	0.132	0.896
Gender	0.139	0.839	0.021	0.166	0.869
Marital status	-1.656	0.981	-0.204	-1.689	0.096
smoking	-0.325	0.838	-0.051	-0.387	0.700

PU, positive urgency; F value, the principal component value of BDNF, PI3K, AKT and CREB; BMI, body mass index.

**Table 10:** Multivariate regression analysis of SS subscale score and influencing factors in patients

	B	SE	β	t	P
F value	-0.530	0.186	-0.349	-2.854	0.006
age	0.001	0.033	0.003	0.024	0.981
BMI	-0.186	0.138	-0.160	-1.346	0.183
Education	0.027	0.105	0.030	0.262	0.794
Gender	1.134	0.784	0.188	1.446	0.153
Marital status	-0.166	0.916	-0.022	-0.182	0.857
smoking	0.072	0.783	0.012	0.092	0.927

SS, sensation seeking; F value, the principal component value of BDNF, PI3K, AKT and CREB; BMI, body mass index.

**Table 11:** Multivariate regression analysis of PR subscale score and influencing factors in patients

	B	SE	β	t	P
F value	-0.356	0.180	-0.252	-1.979	0.052
age	-0.070	0.032	-0.290	-2.187	0.033
BMI	-0.126	0.134	-0.117	-0.947	0.347
Education	0.031	0.101	0.036	0.302	0.764
Gender	-0.206	0.760	-0.037	-0.271	0.787
Marital status	2.129	0.888	0.303	2.398	0.020
smoking	1.160	0.759	0.209	1.529	0.131

PR, lack of premeditation; F value, the principal component value of BDNF, PI3K, AKT and CREB; BMI, body mass index.

**Table 12:** Multivariate regression analysis of PE subscale score and influencing factors in patients

	B	SE	β	t	P
F value	-0.572	0.173	-0.411	-3.303	0.002
age	-0.066	0.031	-0.277	-2.138	0.036
BMI	-0.090	0.129	-0.085	-0.699	0.487
Education	-0.051	0.098	-0.062	-0.525	0.602
Gender	-0.152	0.732	-0.027	-0.208	0.836
Marital status	1.001	0.855	0.144	1.171	0.246
smoking	1.054	0.731	0.193	1.443	0.154

PE, and lack of perseveration; F value, the principal component value of BDNF, PI3K, AKT and CREB; BMI, body mass index.

## Discussion

The depressive symptoms and impulsive behaviors of schizophrenic patients are seen in multiple disease stages. Studies have shown that the BDNF level in the peripheral blood of schizophrenic patients was declined [16]. There was decreased PI3K expression in the animal and cell models of schizophrenia [40, 41], down-regulated PI3K mRNA expression and increased AKT mRNA expression in the peripheral blood of schizophrenic patients [42, 43], and increased susceptibility of schizophrenic symptoms in AKT3-deficient mice [44]. CREB was lowly expressed in the schizophrenic mouse model, while over-expressed CREB could negatively regulate BDNF [26]. Our study results demonstrated that BDNF, PI3K and CREB levels in the patient group were lower than those in the control group, while the AKT level was higher, which is partially consistent with the previous study results. BDNF had a nourishing effect on nerve cells, and its level and mRNA expression were both decreased in the peripheral blood of schizophrenic patients, which indicates possible neuratrophy in the pathogenesis of schizophrenia that induces brain injury [45]. AKT is serine/threonine kinase, and its activity balance is closely related to cell growth, proliferation, survival, and differentiation, thus AKT plays a critical role in maintaining the normal function of the brain. PI3K/AKT is a down-stream intracellular signaling pathway of BDNF. BDNF can promote AKT phosphorylation and thus achieve nerve protection in ischemic brain injury as shown by other studies [46], and the phosphorylation of PI3K and its effector AKT can stimulate CREB activation. CREB is one of the main regulatory factors for the BDNF reaction, phosphorylated CREB binds with the specific sequence of BDNF promoter to regulate the transcription of BDNF and can up-regulate BDNF expression [26]. In our study results, a decrease of PI3K level might be associated with a decline of BDNF level in the upstream, and an increased AKT level perhaps owed to its reduced phosphorylation, followed by weakened activation and resultantly decreased expression of CREB in the peripheral serum. This further suggests that there is a disturbance of BDNF/PI3K/AKT/CREB pathways in schizophrenic patients, with abnormal manifestations of neurotrophy, synaptic transmission, and cellular signaling. However, some previous study results are not coincident with our study results, for example, a study showed that a decrease in AKT level was found in the dorsolateral prefrontal cortex [47]. We believe that the differential changes of protein factors in schizophrenic patients may be associated with the course and severity of the disease, and the nervous system perhaps initiates the mechanisms of “Compensatory Response” and “Decompensatory Response”. These findings suggest that the AKT level increases in the early stage of stress, and such an increase is not kept continuously in the late stage, and then different degrees of clinical symptoms appear.

Pro-BDNF can be transformed into mature-BDNF by intracellular protein hydrolysis in the trans Golgi network via Furin structural release [48], or by extracellular matrix metalloproteinases or fibrinogenase digestion [49, 50]. In the synaptic space, mature-BDNF can bind with the TrkB receptor to promote the homodimerization of the latter and the phosphorylation of tyrosine residue in cells [51, 52]. Phosphorylated TrkB can activate the PI3K signaling pathway and thus facilitate neurogenic, synaptogenic, and other activities [22]. Pro-BDNF can activate the c-Jun N-terminal kinase (c-JNK) pathway by binding with the 75 kDa neurotrophin receptor (p75NTR) at the mature domain and with co-receptors (e.g., sorbin) at the pro-domain, and thus induce programmed cell death [53]. It is thereby seen that the effects of pro-BDNF and mature-BDNF are sometimes opposite. In the subsequent study, we will improve the methodology and validate the measurements of blood pro-BDNF/mature-BDNF levels.

Depression is regarded as an important dimension of psychiatric symptoms [54], but it frequently isn’t given enough sufficient emphasis for similarity to negative symptoms in schizophrenic patients and other reasons [55]. In this study, the incidence rate of depression was 55.56% in drug-naïve patients with first-episode schizophrenia, which is consistent with a view that there is a higher morbidity of depression in patients with first-episode schizophrenia than in those with chronic schizophrenia [56]. However, it was 30-70% in the previous study reports of CDSS-based evaluation on schizophrenic depression [57, 58]. It should be noted that only the patients with first-episode schizophrenia and without the use of antipsychotics were included in our study, which avoids drug interference with the biological mechanism and prevents depression masking. Therefore, in this study, we first reported the incidence rate of depression in drug-naïve patients with first-episode schizophrenia. Many studies have suggested that the BDNF level in the peripheral blood is decreased in patients with schizophrenia [57, 59] or severe depressive disorder [60]. In the present study, the serum BDNF expression level was lower in drug-naïve patients with first-episode schizophrenia than in the healthy control group, and it was smaller in the depressive patient subgroup than in the non-depressive patient subgroup. Our study results showed that there was a significant correlation between the BDNF level and CDSS score in the patient group, which indicates that BDNF may be associated with the pathomechanism of schizophrenia and the occurrence of depression in schizophrenic patients. The PI3K-AKT signaling pathway is one of the numerous pathways shared by schizophrenia and severe depressive disorder [61]. So far, there is no study that explores the serum levels of PI3K and AKT as well as their relations with depressive symptoms in drug-naïve patients with first-episode schizophrenia. We found that the serum PI3K level in the patient group was significantly lower than that in the healthy control group, which is partially coincident with the previous study results that the serum PI3K mRNA level in schizophrenic patients was smaller than that in the healthy control group but the AKT mRNA level was greater [42]. Besides, the PI3K level was much lower in patients with significant depressive emotion than in those with non-significant depressive emotion. However, the results of AKT and PI3K were opposite. We also observed that depressive emotions were negatively correlated with both PI3K and AKT expression levels. In other studies of central nervous system diseases, it was found that phosphorylated AKT or the total AKT level in the peripheral blood was higher in patients than in the healthy control group and had a positive correlation with the disease duration and severity [62]. It indicates that the peripheral expression change of specific kinases is of certain significance for the research of nerve degeneration. In the depressive mice study, CREB/BDNF expression was restored by anti-inflammatory treatment, and then depressive symptoms were relieved [63]. This is consistent with our study results of CREB, which suggests that CREB may participate in the depression development of schizophrenic patients through similar inflammatory reactions and nerve plasticity. In another study, it was observed that CREB activity in the lymphocytes and BDNF level in the plasma of peripheral blood were associated with Alzheimer’s disease, meanwhile, CREB activity and BDNF level in platelets seemed to be related with to depression [64]. In patients with Alzheimer’s disease, phosphorylated CREB expression in the monocytes of peripheral blood was positively correlated with that in the brain after death; it indicates that CREB signaling dysfunction may be not only limited to the brain, and there is a possible linkage of CREB regulation between blood and brain [65]. The above studies have suggested that the blood CREB level can reflect the brain to a certain degree, and has certain significance in the diseases of the central nervous system.

Our study revealed that the schizophrenic patients had obvious impulsive behaviors, and the phosphorylated CREB level in the brain was increased in the previous mouse study of dopamine receptor gene knock-out and drug-induced cognitive impairment and impulsive behaviors [66], which suggests that CREB is involved in regulating impulsive behaviors via different pathways. Our study showed that the expression levels of BDNF, PI3K, AKT, and CREB were significantly correlated with S-UPPS-P score, indicating that BDNF, PI3K, AKT, and CREB not only participate in the development of schizophrenia, the expression level of the PI3K-AKT signaling pathway can also be used as a state indicator of schizophrenic patients. Interestingly, our study results demonstrated that BDNF, PI3K, and CREB were in a mutually positive correlation, and AKT was negatively correlated with the former three; these four serum protein factors had significant interactions in CDSS and S-UPPS-P scores of schizophrenic patients and further played their functions. Furthermore, previous studies have shown that these four proteins and their classic pathways are closely related to schizophrenia, so the conclusion is more persuasive that there are correlations among the four proteins. However, considering such high correlations, the factor of no overall representativeness due to a small sample size cannot be completely excluded.

Our study has some limitations. This is a case-control study that does not support clarifying the causality between the indicators of serum protein factors and the depressive symptoms and impulsive behaviors in schizophrenic patients. Although only drug-naïve patients with first-episode schizophrenia were included in the patient group, the course of the disease was still heterogeneous, and reasonable stratification based on the course of the disease will be considered in the future. In our study, there were a limited number of cases included, and only the cross-sectional data were analyzed. In the subsequent study, we will increase the sample size, carry out a further longitudinal investigation, properly add some measures of reducing the experimental errors, and follow up on the improvement of depressive emotion and impulsive behaviors in patients.

## Conclusion

Our study results have suggested that schizophrenic patients have abnormal manifestations of neurotrophy, synaptic transmission, and cellular signaling; BDNF, CREB, PI3K, and AKT are not only associated with schizophrenia, but also correlated with depressive symptoms and impulsive behaviors in schizophrenia to different degrees; the interactions among serum protein factors may aggravate depression and impulse. However, these findings shall be validated in more detailed pathway studies and longitudinal studies. In the present study, our major subjects were drug-naïve patients with first-episode schizophrenia, and we found that the relevant serum protein factors might help predict the clinical symptoms such as depression and impulse, and thus provide a possibility of identifying the biomarkers of different symptom dimensions of schizophrenia.

## Data Availability

The datasets presented in this article are not readily available because the data are currently part of a longitudinal study that is ongoing. However, they can be obtained by contacting the corresponding author. Requests to access the datasets should be directed to corresponding author xp6945399@163.com.

## Abbreviations

BDNF: brain-derived neurotrophic factor
PI3K: phosphatidylinositol-3 kinase
AKT: protein kinase B
CREB: cAMP response element binding protein
CDDS: Calgary Depression Scale for Schizophrenia
S-UPPS-P: the Short UPPS-P Impulsive Behavior Scale
ELISA: Enzyme Linked Immunosorbent Assay
BMI: body mass index
PANSS: Positive and Negative Syndrome Scale
PIP2: phosphatidylinositol 4,5-biphosphate
PIP3: phosphatidylinositol 3,4,5-trisphosphate
PTEN: phosphatase and tensin homolog
PDK1: phosphoinositide dependent kinase 1
SSD: schizophrenia spectrum disorders
TrkB: tropomyosin-related kinase B
MAPK: mitogen-activated protein kinase
PLC-γ: phospholipase C-γ
p75NTR: 75 kDa Neurorophin Receptor
NU: negative urgency
PU: positive urgency
SS: sensation seeking
PR: lack of premeditation
PE: lack of perseveration
